# CT Evaluation of Hematuria in Adults Younger than 50 Years in Military Service: Is Contrast-Enhanced Phase Needed?

**DOI:** 10.3390/jcm14124051

**Published:** 2025-06-08

**Authors:** Gil N. Bachar, Inna Tsitman, Nir Popel, Shahar Porat, Tomer Erlich, Eli Atar

**Affiliations:** 1 Faculty of Medicine, Tel Aviv University, Tel Aviv 6997801, Israel; elia@clalit.org.il; 2 Department of Diagnostic Imaging, Rabin Medical Center, Petah Tikva 4941492, Israel; innatsitman@gmail.com; 3 Department of Diagnostic Imaging, Medical Corps, Israel Defense Force, Ramat Gan 5211200, Israel; popelnir@gmail.com (N.P.); shahar.porat@gmail.com (S.P.);

**Keywords:** hematuria, CT urography, military, young adults

## Abstract

**Background:** Limited data exist on the comparative diagnostic value of CT urography (CTU) versus unenhanced CT in evaluating the upper urinary tract in young adults (<50 years) with hematuria in active military service. This population may face an increased risk of urinary tract malignancies due to occupational exposures. **Methods**: We conducted a retrospective cohort study of 277 consecutive Israel Defense Forces personnel under 50 years old with new-onset hematuria referred for CT evaluation between 2011 and 2020. Two experienced radiologists first interpreted unenhanced CT images, followed by a review of contrast-enhanced phases. Findings were classified based on their detectability on unenhanced CT and whether contrast phases were required. **Results**: Of the 277 patients, 270 had microscopic hematuria and 7 had macroscopic hematuria. Imaging was normal in 158 cases. Among 119 patients (43%) with positive findings, 46 (16.6%) had clinically significant findings requiring follow-up or treatment. Of these, 42 (91%) were detectable on unenhanced CT alone. Contrast phases were requested in 15 cases (5.4%) and revealed additional benign findings. No urinary tract malignancies were identified. **Conclusions**: Unenhanced CT may be sufficient for evaluating new-onset hematuria in adults under 50, including active military personnel, minimizing the need for contrast administration.

## 1. Introduction

Hematuria is a prevalent urologic finding in adults, with an estimated prevalence ranging from 2.5% to 38.7% in population-based screening studies [1,2]. It can present in two forms, including macroscopic hematuria, which is observable without the aid of a microscope, and microscopic hematuria, which is characterized by the detection of more than three red blood cells per high-power field in a urine sample.

Potential causes of hematuria include renal calculi, infections, trauma, renal parenchymal disease, recent instrumentation, prolonged exercise, drug toxicity, coagulopathy, and neoplasms [3]. However, hematuria is frequently a benign incidental finding, with the etiology remaining unknown in approximately 70% of patients. Urinary malignancy is identified in only 0.4% to 3.4% of patients [4,5,6].

The American Urological Association and the American College of Radiology recommend computed tomography urography (CTU) in conjunction with cystoscopy of the urinary bladder and urine cytology as the preferred modality for evaluating new-onset microhematuria in adults, without age stratification [7,8]. Chlapoutakis et al. [9] conducted a meta-analysis on the efficacy of CTU for detecting urothelial malignancy, confirming its high sensitivity and specificity compared to intravenous pyelography. However, several studies have indicated that the diagnostic benefit of CTU over unenhanced CT is limited in younger adults (under 50 years) with asymptomatic hematuria [10,11,12]. Moreover, considering the radiation exposure associated with CTU and the necessity for iodine contrast injection, the Dutch Association of Urology has recommended ultrasound as the preferred study in young adults, where the incidence of urinary malignancy is relatively low [12,13].

Nonetheless, certain adult patient groups are at a heightened risk for urinary malignancies compared to the general population, such as military veterans who may be exposed to potential carcinogens including metallic materials, aircraft machinery, and specific toxic substances associated with urinary malignancy [10,14]. Sohn et al. [15] reported that the rates of kidney and bladder cancer increased by 35% and 33%, respectively, among U.S. military veterans over a three-year period. Smoking is recognized as the most significant risk factor for urinary malignancies [14]. A study conducted by Zarka et al. [16] involving 30,000 Israeli Defense Forces (IDF) soldiers from 1987 to 2017 indicated that active smoking rates increased by 39% during compulsory military service, rising from 26.2% at recruitment to 36.5% at discharge.

The objective of the present study was to compare the diagnostic yield of CTU with unenhanced CT in soldiers aged under 50 years who present with hematuria.

## 2. Materials and Methods

### 2.1. Patients

The study was conducted according to the guidelines of the Declaration of Helsinki and approved by the local Institutional Review Board of the Chief Medical Corps of the IDF (IRB 1988-2019, Version 3), with the date of approval as 30 June 2019. Patient consent was waived by the institutional review board due to the retrospective design of the study and the use of anonymized data. The study group comprised 277 consecutive patients under the age of 50 years who were actively serving in the IDF. These patients were diagnosed with new-onset hematuria by a military urologist between January 2011 and March 2020 and were subsequently referred to our CT unit for evaluation of the upper urinary tract. The exclusion criteria included individuals over the age of 50 years, those with a history of nephrolithiasis, a history of urological malignancy, and recurrent urinary tract infections.

### 2.2. CTU Protocol

CT imaging was conducted using a 64-slice multidetector scanner with specific settings including a tube voltage of 120 kV and a quality reference tube current-time product of 100 mAs. The scans were performed during inspiratory breath-holding, with a collimation of 64 mm × 1.5 mm and a reconstruction slice width of 3 mm, without the use of oral contrast or bowel preparation. A 100 mL injection of a nonionic contrast agent was administered via a dual-head automatic injector, followed by the acquisition of 3 mm reconstructed axial images in three phases, namely non-contrast, nephrographic, and excretory. Additionally, coronal and sagittal reconstructed images were sent to a picture archiving and communication system (PACS).

### 2.3. Image Analysis

All examinations, including axial and multiplane reconstructions, were reviewed on the PACS workstation by one of two board-certified abdominal radiologists with 10 years of experience (E.A. and T. I.). The radiologists were blinded to the patients’ clinical histories but were aware that the purpose of the imaging study was the initial evaluation of hematuria. For each study, the reader first interpreted the unenhanced images and recorded all the urological and other findings. The reader then reviewed the nephrographic and excretory phases with different window levels for an evaluation of the renal parenchyma, calyces, and collecting system ureters and recorded all urological findings. The findings detected only on contrast-enhanced images were tabulated. If no urological findings were detected on either the unenhanced examination or the enhanced series, the study was evaluated by a third board-certified abdominal radiologist (B.N.G.) with 20 years of experience (the adjudicator) who was also blinded to the clinical interpretation and clinical history of the patient. The adjudicator characterized any findings visible on non-contrast CT alone or on the requested contrast-enhanced images.

### 2.4. Diagnosis

Demographic variables, including age and sex, medical history, reason for referral, pathology tests, and radiological evaluations such as ultrasound and MRI, were meticulously recorded for each patient in an electronic database. Additionally, further urologic work-ups, including cystoscopy, ureteroscopy, and biopsy, were conducted, culminating in a final diagnosis. In instances where the work-up yielded negative results, clinical follow-up was undertaken, involving a comprehensive review of the patient’s medical records to ascertain whether any urinary tract abnormalities were subsequently diagnosed.

## 3. Results

Table 1 shows consecutive patients presenting with new-onset hematuria, including 229 men (82.7%) and 48 women, with a mean age of 30.44 ± 10.3 years. Micro-hematuria was diagnosed in 270 patients, while macrohematuria was identified in seven. The mean follow-up duration was 40 months, ranging from 6 to 86 months.

Among the patients with positive CTU results, 46 (16.6%) presented with clinically significant findings necessitating treatment (as shown in Table 2). These findings were observable on unenhanced images in 42 patients (91%). The predominant findings included urinary tract calculi, involving renal (*n* = 32), ureteral (*n* = 4), and bladder (*n* = 1) calculi. Notably, one patient exhibited a pre-sacral multi-lobulated mass measuring 5 cm× 4 cm, consistent with a tailgut duplication cyst. Another patient, who presented with microscopic hematuria, had a 3 mm polyp that was resected via cystoscopy and was confirmed to be an inverted papilloma of the urinary bladder. In the remaining four patients, significant findings were detected exclusively on contrast-enhanced images. Three of these patients, all experiencing recurrent microscopic hematuria and flank pain, displayed striated nephrograms attributed to pyelonephritis, and they received antibiotic treatment. Follow-up evaluations, including cystoscopy and magnetic resonance imaging, yielded normal results. The fourth patient, aged 35 years, had previously undergone nephrectomy for a posterior urethral valve in childhood. CT imaging revealed hydronephrosis and hydroureter, while contrast-enhanced CT indicated reflux.

Additionally, 21 patients exhibited clinically significant findings that warranted observation alone. Two patients presented with atrophic kidneys, and two others had hyperdense complicated cysts, which were enhanced on CT and were consistent with hemorrhagic cysts (Bosniak 2F); these patients were monitored with magnetic resonance imaging over a five-year period. In three patients, a thickened and trabeculated bladder wall was observed in excretory phase images, although it was not visible on unenhanced images when the bladder was under-distended. Cystoscopy results were normal for all cases. Two patients had a thickened and enhanced distal ureter, with normal findings on ureteroscopy. A left uretrocele was identified in one patient with polycystic kidney disease, while another patient had a thickened appendix (up to 13 mm) with peri-appendicular straining, leading to a diagnosis of appendicitis, which was managed conservatively (refer to Table 1).

Contrast images were requested by the readers in 15 cases (5.4%) to confirm and characterize benign findings. These included pyelonephritis, trabeculated and thickened bladder wall, renal scars (*n* = 3 each), complicated renal cysts (Bosniak 2F), thickened and enhanced distal ureters (*n* = 2 each), and a tailgut duplication cyst with reflux in a single kidney (*n* = 1 each).

Clinically insignificant findings were detected in 67 patients, including benign renal cortical cysts in 29 patients (21 Bosniak 1, 6 Bosniak 2, 1 Bosniak 2F), all of which were visible on unenhanced images. Additionally, a double collecting system was identified in 12 patients (unilateral in 10, bilateral in 2), uretero–pelvic junction obstruction in 12 (also visible on unenhanced images), and benign prostate hypertrophy in three patients. Urachal diverticulum, urachal cyst, urachal sinus, pelvic kidney, and horseshoe kidney with uretero–pelvic junction obstruction were detected in one patient through unenhanced imaging.

An adverse reaction to intravenous iodinated contrast material was reported in two of the 277 patients (0.7%), presenting as a rash and shortness of breath.

The CT scanner measured the effective radiation dose that patients were exposed to, with the average dose per patient varying between 18 and 25 mSv.

## 4. Discussion

This study sought to determine whether the concerns regarding the appropriateness of CTU for adults aged less than 50 years with hematuria raised by the Dutch Association of Urology [12,13] and others [11,17] also hold true for members of this age group with particular risk factors for urinary malignancy.

CTU has been found beneficial for the evaluation of the urinary tract in adults with persistent hematuria and is recommended for this purpose by the American College of Radiology [8]. In 2010, Chlapoutakis et al. [9] reviewed and analyzed the published literature and found that CTU had a pooled sensitivity of 96% and specificity of 99% for the detection of urothelial malignancies. Chow et al. [18], in a study of 500 adult patients, reported corresponding rates of 100% and 99%, with positive and negative predictive values of 80% and 100%, respectively. However, when the population was restricted to adults aged <50 years with asymptomatic hematuria and no risk factors, two studies found that although CTU had diagnostic benefits, unenhanced CT alone could serve as an efficient diagnostic modality [3,11]. However, no study has assessed the added value of CTU in high-risk young adults.

In the present study, CTU was used to evaluate patients aged 18–50 years with hematuria in active military service and hence at increased risk of urological malignancies due to occupational exposure [10]. Moreover, running and strength training are common military practices, and many studies have shown a link between intense exercise and hematuria. Siegel et al. [19] found that 18% of 50 marathon runners with normal pre-race samples had microscopic hematuria, and Jones et al. [20] reported a 90% rate of post-workout hematuria in middle-distance track athletes. In a retrospective study of 1000 young individuals (age < 40 years) in the Israeli Air Force, Froom et al. [21] found a 38% prevalence of asymptomatic hematuria over a 15-year period.

In the present study, more than half of the evaluated patients with hematuria had no upper urinary tract abnormalities on CT. This high rate of negative findings is in accordance with other studies [2,10,11] and is probably a consequence of the young age of the patients and the likelihood of exercise-induced hematuria. This suggests that CTU may be inappropriate as the first-line investigation for asymptomatic hematuria in this population because it may expose healthy young adults to unnecessary radiation and contrast medium.

Furthermore, among the 119 patients (43.0%) who underwent a positive urological study, the findings were clinically insignificant in 73 (61.3%), consisting of benign renal cortical cysts, double collecting systems, and uretero–pelvic junction obstructions. All insignificant findings were detected on unenhanced CT scans. The remaining 46 patients (16.6%) showed clinically significant findings, mainly renal or ureteral calculi, which were also detected in unenhanced CT scans. Contrast images were requested by the readers in only 15 cases (5.4%) to confirm and characterize benign findings, including pyelonephritis, renal complicated cysts, thickened and trabeculated bladder wall, renal scar, thickened and enhanced distal ureter, tailgut duplication cyst (one patient), and reflux in a single kidney (one patient).

Given that the majority of clinically significant and insignificant findings in our study were evident on unenhanced images, we suggest that most young adults with hematuria should be optimally evaluated with unenhanced CT, reserving CTU for patients with persistent hematuria or other predisposing conditions.

Importantly, we found that CTU did not detect renal or urothelial carcinoma in any of the patients with hematuria.

CT urography (CTU) was used for young adults presenting with hematuria. In particular, this is comparable to studies by Mace et al. [10] and Lokken et al. [11], which also investigated the diagnostic yield of CTU in young populations, including military cohorts.

Mace et al. [10] assessed 137 U.S. military personnel under 50 years of age and found that the majority of urological findings were either benign cysts or nephrolithiasis, with no malignancies detected. Contrast-enhanced phases were requested in only 14 cases, all to characterize benign findings. These results led the authors to suggest that unenhanced CT may suffice for initial evaluation, even in higher-risk groups.

Similarly, Lokken et al. [11] evaluated 375 young adults (≤40 years) and reported that 22.1% had clinically significant findings, of which 94.8% were detectable on unenhanced images. Importantly, the few findings that required contrast enhancement were all found in patients with predisposing medical conditions. Four malignancies were identified, reinforcing the need for a selective use of contrast-enhanced imaging.

Mishriki et al. [22], in a prospective study, found no malignancies in 292 patients in the same age group referred for the evaluation of asymptomatic microscopic hematuria and followed-up for 13 years. According to epidemiologic studies, the average age at diagnosis of urological malignancy is 65 years [10], suggesting that older age (more than 50 years) is a risk factor [2,10] for cancer-associated hematuria.

Young age may also explain the lack of urological malignancy in seven patients (2.7%) with gross hematuria, a significant risk factor for urological malignancies and life-threatening disease. In a retrospective study of 1209 patients with hematuria, Song et al. [23] found an eight-fold increase in malignant diagnoses in those with gross hematuria (18.4%) compared to those with microhematuria (2.3%). Similar findings have been reported previously [24,25].

This study offers several strengths compared to previous investigations. It includes a relatively large homogenous cohort of 277 active-duty military patients under the age of 50—a population with distinct occupational exposure risks but without known medical comorbidities. In contrast to earlier studies with smaller or more heterogeneous samples, this targeted population enhances the relevance of our findings.

We applied a clinically meaningful classification of CTU findings into three categories as follows: requiring treatment, requiring observation, or clinically insignificant. This stratification facilitates a clearer interpretation of CT utility in young adults with hematuria. Notably, 91% of clinically significant findings were visible on unenhanced CT, and contrast was required in only 5.4% of cases—primarily to confirm benign lesions.

Furthermore, the extended follow-up (mean 40 months) adds confidence in the safety of a conservative imaging strategy. These results support the use of unenhanced CT as a sufficient initial modality in most cases.

Radiation-induced cancer rates vary widely in the medical literature. According to some studies, approximately 1.5–2% of all cancer cases in the US are attributable to radiation [26]. In addition, CTU studies include multiple phases requiring an increased radiation dose, which has been reported as 25–35 mSv compared with 3–10 mSv for a single unenhanced CT study [23,27]. A low-dose unenhanced technique can further reduce radiation to 1.4–2.1 mSv [2,28]. In the present study, had CTU not been used, 95% of patients (262/277) would have been spared a mean effective radiation dose of 25–35 mSv or a total of 6550 mSv for the whole study population.

An adverse reaction to intravenous iodinated contrast material was documented in 2 of our 277 patients (0.7%), manifesting as rash and shortness of breath. The estimated risk ranges from 0.3% to 1.6%, with reported manifestations including hives, bronchospasms, laryngeal edema, contrast-induced nephropathy, and even death [2,29].

Overall, our findings raise the question of whether CTU is the optimal modality for the evaluation of hematuria in young adults. This patient population had a low likelihood of malignancy and a high prevalence of urinary tract calculi. This is especially true for individuals with active military duty, among whom exercise-induced hematuria is common. In terms of imaging modality, CTU poses an increased risk of radiation-related complications, allergic reactions to contrast medium, contrast extravasation, and nephropathy. This can be avoided using non-contrast low-dose CT or ultrasound. Accordingly, ultrasound is recommended as the primary imaging modality for the evaluation of hematuria by the European Society of Urogenital Radiology for patients younger than 40 years [30] and by the Dutch Association of Urology for patients younger than 50 years [13].

This study had several limitations. The study was performed at a single center using a retrospective design and was based on data only from patients who underwent CTU because of asymptomatic hematuria. Furthermore, although the follow-up period was sufficiently long (40 months), we have limited the long-term follow-up of patients whose CTU examination results were normal.

In conclusion, we found that non-contrast CT may be more appropriate than CTU as a first-line investigation for the evaluation of asymptomatic hematuria in patients younger than 50 years, including those in military service.

## Figures and Tables

**Table 1:** Characteristics of 277 military personnel with hematuria.

Characteristics	Number
Gender	
Male	229 (82.7%)
Female	48 (17.3%)
Age (years), mean ± SD (%)	
≤40	210 (75.8%)
41–50	67 (24.2%)
Type of hematuria	
Microhematuria	270 (97.4%)
Macrohematuria	7 (2.6%)

Positive and negative findings from computed tomography urography (CTU) were recorded in 119 (43.0%) and 158 (57.0%) patients, respectively (refer to Table 2). Importantly, none of the patients were found to have a urological malignancy.

**Table 2:** Findings on CT urography in patients less than 50 years old in active military service with hematuria.

Diagnosis	No. of Patients
**Significant findings**	
Nephrolithiasis	
Renal calculus	32
Ureteral calculus	4
Bladder calculus	1
Pyelonephritis	3
Appendicitis	1
Tailgut duplication cyst	1
Inverted papilloma of urinary bladder	1
Hydroureter and reflux	1
Ureterocele	1
Polycystic kidney disease	1
**Insignificant findings**	
Duplicated collecting system	
Right	4
Left	6
Bilateral	2
Renal cysts	
Bosniak category 1	21
Bosniak category 2	6
Bosniak category 2F	2
Uretero–pelvic junction obstruction	12
Benign prostate hypertrophy	3
Urachal diverticulum	1
Urethral cyst	1
Urachal sinus	1
Atrophic kidney	2
Renal parenchymal scarring	3
Pelvic kidney	1
Horseshoe kidney	1
Agenesis of left kidney	1
Renal angiomyolipoma	1
Diverticula of bladder	1
Thickened and trabeculated bladder	3
Left varicocele	1

## Data Availability

Data is unavailable due to privacy and ethical restrictions.

## Abbreviations

CT	Computed tomography
CTU	CT urography
IDF	Israel Defense Forces
PACS	Picture archiving and communication system
